# Using and Reporting the Delphi Method for Selecting Healthcare Quality Indicators: A Systematic Review

**DOI:** 10.1371/journal.pone.0020476

**Published:** 2011-06-09

**Authors:** Rym Boulkedid, Hendy Abdoul, Marine Loustau, Olivier Sibony, Corinne Alberti

**Affiliations:** 1 AP-HP, Hôpital Robert Debré, Unité d'Epidémiologie Clinique, Paris, France; 2 Inserm, CIE 5, Paris, France; 3 AP-HP, Hôpital Robert Debré, Pôle Gynécologie et périnatalité, Hospitalisation Gynécologie – Obstétrique, Paris, France; 4 Université Paris 7 Denis Diderot, UFR de Médecine, Paris, France; University of British Columbia, Canada

## Abstract

**Objective:**

Delphi technique is a structured process commonly used to developed healthcare quality indicators, but there is a little recommendation for researchers who wish to use it. This study aimed 1) to describe reporting of the Delphi method to develop quality indicators, 2) to discuss specific methodological skills for quality indicators selection 3) to give guidance about this practice.

**Methodology and Main Finding:**

Three electronic data bases were searched over a 30 years period (1978–2009). All articles that used the Delphi method to select quality indicators were identified. A standardized data extraction form was developed. Four domains (questionnaire preparation, expert panel, progress of the survey and Delphi results) were assessed. Of 80 included studies, quality of reporting varied significantly between items (9% for year's number of experience of the experts to 98% for the type of Delphi used). Reporting of methodological aspects needed to evaluate the reliability of the survey was insufficient: only 39% (31/80) of studies reported response rates for all rounds, 60% (48/80) that feedback was given between rounds, 77% (62/80) the method used to achieve consensus and 57% (48/80) listed quality indicators selected at the end of the survey. A modified Delphi procedure was used in 49/78 (63%) with a physical meeting of the panel members, usually between Delphi rounds. Median number of panel members was 17(Q1:11; Q3:31). In 40/70 (57%) studies, the panel included multiple stakeholders, who were healthcare professionals in 95% (38/40) of cases. Among 75 studies describing criteria to select quality indicators, 28 (37%) used validity and 17(23%) feasibility.

**Conclusion:**

The use and reporting of the Delphi method for quality indicators selection need to be improved. We provide some guidance to the investigators to improve the using and reporting of the method in future surveys.

## Introduction

The Institute of Medicine defines healthcare quality as “the degree to which health services for individuals and populations increase the likelihood of desired health outcomes and care consistent with current professional knowledge” [1]. Improving the quality and safety of healthcare has generated considerable attention in recent years [2]. As part of this thrust, authorities and health care professional used a wide range of methods and tools to promote quality improvement. During the past decade, the development and implementation of quality indicators (also known as performance indicators or quality measures) has been largely driven by the arrival of computerised administrative and clinical database and the desire to make performance data available publicly [3]. Many governmental associations and professional bodies have developed quality indicators for different areas in order to improve the quality of care and detect suboptimal care either in structure, process or outcome [4].

The information required to develop quality indicators can be obtained using systematic or nonsystematic methods. Nonsystematic approaches such as case studies are based on data availability and real-time monitoring of critical incidents [5]. Although these approaches play an important role, they fail to exploit much of the available scientific evidence. In systematic approaches, in contrast, indicator selection relies directly on the available evidence, complemented when necessary with expert opinion [6], [7]. Experts examine the evidence and reach a consensus. Systematic methods enhance decision making [8]; facilitate the development of quality indicators or review criteria for areas where the evidence alone is insufficient [9] or controversy [10],[11]; and synthesize accumulated expert opinion. Among these methods, the Delphi technique has been widely used for quality-indicator development in healthcare.

The Delphi technique is a structured process that uses a series of questionnaires or ‘rounds’ to gather information. Rounds are held until group consensus is reached [10], [12]. One of the main reasons for the popularity enjoyed by the Delphi technique is that a large number of individuals across diverse locations and areas of expertise can be included anonymously, thus avoiding domination of the consensus process by one or a few experts [13]. Adler et al. [14] defined the Delphi technique as an exercise in group communication that brings together and synthesizes the knowledge of a group of geographically scattered participants who never meet.

The Delphi technique is among the methods used to develop prescribing indicators [9], indicators reflecting patient and general practitioner perspectives of chronic illness [15], performance indicators for emergency medicine [16], and indicators for cardiovascular disease [17]. Currently, there are no universally accepted requirements for using the Delphi technique [8]. Considerable confusion, disagreement, and uncertainty exist concerning the parameters of the Delphi technique such as the definition of group consensus, Delphi technique variants, expert selection, number of rounds, and reporting of the method and results [18].

The main objective of this study was to describe and discuss the use of the Delphi technique for quality indicator selection and to assess the reporting of the method and results. We sought to identify specific methodological criteria regarding the use of Delphi techniques for quality indicator selection. Finally, we developed a number of best-practice guidance.

## Methods

### Article Selection

We searched Medline via PUBMED, EMBASE and COCHRANE library using the search terms “Delphi” AND “Healthcare”, with no date limits. We chose these broad terms because using restrictive terms might have failed to retrieve all the articles of interest. We identified all reports of studies in which Delphi techniques were used to select quality indicators.

Retrieved articles were assessed by one of us (RB), who read the titles and abstracts to identify the relevant studies. Articles were included only if the study assessed the use of Delphi techniques to select quality indicators in healthcare and was published as a full-text article. We excluded studies reported only in abstract form, editorials, methodological studies, comments, and duplicate publications.

A further search was conducted in both PUBMED and EMBASE using the more specific terms “Quality Indicators, Health Care”[Mesh]) AND (“Delphi Technique”[Mesh]). We then compared the results of the two search strategies.

To evaluate the use and reporting of Delphi techniques and results, we developed a standardized data extraction form (**APPENDIX S1**). The items in the form were selected based on information from articles identified through a literature search [6], [8], [19]. These items pertained to preparation of the Delphi questionnaire, selection of the experts, characteristics of the survey, and reporting of the results.

Before the study, two of us (RB and ML) independently evaluated 10 articles taken at random among the articles selected for the study. They met to discuss the interpretation of the items and to resolve any differences in scoring. Global reproducibility was high, with a median κ of 1(Q1: 0.8, Q3:1).

One of us (RB) recorded the data from each selected article on the standardized data extraction form. For each article were recorded: date of publication, name of the journal and medical specialty of the study.

In addition, the following data were extracted.

### Data on Delphi Questionnaire Preparation

Quality indicators were divided into three categories based on whether they related to structure, process, or outcome. Structure refers to static or technical aspects of care (e.g., attributes of service providers or of the healthcare institution). Process refers to steps taken in caring for the patient and outcome to the impact of care on the health status of patients or populations [20].

We recorded the method used for indicator selection which were include in the first questionnaire and the criteria used to select indicators in each round. We checked whether the article included a clear definition of the selection criteria and/or the definition used in the Delphi questionnaire. We also recorded whether the selection criteria used in the first round were the same as those used in the next round. We extracted the number of quality indicators at the beginning of the survey and we determined whether the experts could add indicators they felt deserved evaluation in subsequent rounds.

### Data on the Expert Panel Size and Composition

For each selected article, we recorded the number of experts invited to participate and whether these experts were first asked about their willingness to participate. We recorded the data supplied in the article about the experts (i.e., specialty, age and years of experience), the composition of the panel (e.g., patients, informal care providers, healthcare professionals, managers), and whether the panel included professionals from a single specialty or from multiple specialties. We determined how the experts were chosen (e.g., willingness to participate, expertise, or membership in an organization). We evaluated the relationship between the response rate and the use of specific methods to encourage the experts to respond (e.g., stamped addressed envelope for returning the questionnaire and financial compensation).

### Data on Progress of the Survey

We evaluated the type of Delphi technique used in each study. We defined the basic Delphi technique as any type of self-administered questionnaire with no meetings and modified Delphi techniques as the combined use of a self-administered questionnaire and of a physical meeting of the experts to discuss the results or rate the indicators [21], [22]. When a modified Delphi technique was used, we determined whether the meeting was held before, after, or between Delphi rounds and what the participants did during the meeting. We recorded the number of rounds. For the basic Delphi method, each round consisted in the completion of a structured questionnaire with the goal of achieving a consensus. For modified Delphi methods, in addition to questionnaire-based rounds, the physical meeting was counted as a round. The time taken to complete the Delphi procedure was recorded, as well as the geographic scope of the survey. We recorded the main methods used to send the questionnaires (e.g., mail, E-mail, or fax). For each study, we checked the formulation of the questionnaire items (e.g., open questions, rating of quality indicators, or both) and whether the quality indicators were rated (in which case, we recorded the minimum and maximum values on the rating scale). We recorded the method used to define a consensus among panel members, whether the percentage of agreement was determined, and whether a cut-off (e.g., median value) was used to select indicators.

We evaluated the methods used by the Delphi procedure organisers to send the responses back to the panel. More specifically, we determined whether the experts were informed of both the response of the group and their own individual response (individual feedback) to each item. For each study, we recorded the type of feedback, which was defined as qualitative when a summary of the panel's comments was sent to each participant and quantitative when simple statistical summaries illustrating the collective opinion (e.g., central tendency and variance) were sent to each participant.

### Data on Delphi Results

We recorded the number of quality indicators selected at the end of the Delphi procedure. We searched each article for a list of quality indicators and, when such a list was found, we determined whether it included all the quality indicators used for the first round or only the indicators selected at the end of the last round. We looked for a flow chart of quality indicators (figure showing the output and input indicators at each round) and/or for a written description of indicator flow, as well as the availability of the questionnaires in the article itself or in an appendix. Finally, we recorded the response rate for each round if available.

### Statistical Analysis

We computed the medians and the first and third quartiles for continuous variables and the number (%) of articles for categorical variables. Percentages for each characteristic were computed using the total number of articles reporting that characteristic as the denominator. Statistical analyses were performed using SAS version 9.1 (SAS Institute Inc, Cary, NC, USA).

## Results

**Figure 1:**
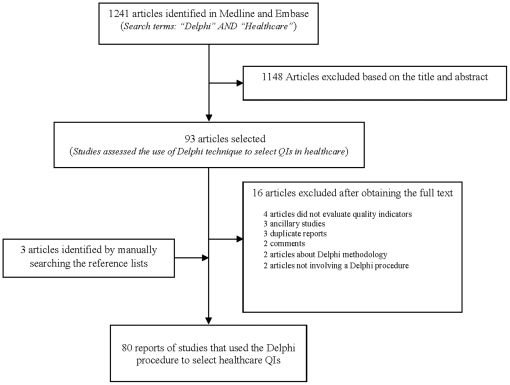
Study selection.

Of the 1241 articles retrieved by our database search, 91 were selected based on the titles and abstracts (**FIGURE 1**); of these, 80 were included in the final analysis. The included articles are described in **TABLE 1**. All were published between 1978 and 2009; however, most of them (n = 64, 80%) were published recently, ie after 2000 (**FIGURE S1**). The research strategy based on restrictive terms retrieved the same articles.

**Table 1:** Characteristics of the 80 selected articles.

Characteristic	
***Journal type***	
Specialty	55 (69)
General medical	15 (19)
Health quality	10 (12)
***Top five medical areas***	
Oncology	10 (13)
Geriatric/end of life/long-term care	10 (13)
Cardiology	8 (10)
Prescribing drugs	8(10)
Mental health/Parkinson/Memory	6 (8)

### Delphi Questionnaire Preparation

**TABLE 2** summarizes the characteristics of the Delphi questionnaires. Methods used to select quality indicators to prepare Delphi questionnaire was reported in 96%(77/80) of studies and criteria used to select indicators during the survey in 94% (75/80). The most often used criteria were validity (28/75, 37%) and feasibility (17/75, 23%). However, a substantial proportion of studies used their own selection criteria (**TABLE S1**).

**Table 2:** Characteristics of the first Delphi questionnaire.

Characteristics	Reported^a^n (%)	Present^b^n (%)
***Type of quality indicators (QIs) selected*** c	**51 (64)**	
Outcome		35 (69)
Structure		24 (47)
Process		41 (80)
Other		2 (4)
**Methods* used to select quality indicators for the questionnaire*** c	**77(96)**	
Literature review		48 (62)
Guidelines		20 (26)
Focus groups		17 (22)
QI developed in another country		11 (14)
Preliminary preparation work^d^		26(32)
***Number of QIs included in the first Delphi questionnaire***	**70 (88)**	
*Median (Q1;Q3)*		59 (32;146)
*Min-Max*		11–767
***Selection criteria*** c	**75(94)**	
Validity		28 (37)
Feasibility		17 (23)
Importance		13 (17)
Agreement or reability		12 (15)
Other (Table S1)		44 (55)
***Number of selection criteria used***		
One		51 (68)
Two		17 (23)
Three		7 (9)

a Percentage of studies that reported this item over the total number of studies (n = 80).

b All percentages were calculated relative to the total number reported.

c The total percentage may exceed 100% because some studies used more than one criterion.

d included Previous work , Internal consensus , Results of first Delphi round, Development of new QI.

Among articles that listed the indicator selection criteria, only 61/75 (81%) clearly defined these criteria. Examples of definition are given in **BOX S1**.

Selection criteria changed between rounds in 13/73 (18%) studies. For example, in one study, indicators were selected based on “applicability” in the first round and based on “validity” and “importance” in subsequent rounds. Only 31/70 (44%) studies allowed the experts to add indicators during the Delphi procedure.

### Characteristics of the Delphi Participants (TABLE 3)

**Table 3:** Description of the Delphi panels.

Characteristics	Reported^a^n (%)	Present^b^n (%)
***Number of individuals invited to participate***	**76 (95)**	
*Median (Q1;Q3)*		17 (11;31)
*Min;Max*		3;418
***Criteria used to choose potential participants*** c	**63 (79)**	
Renown		27 (43)
Member of organization		22 (35)
Recommendation		10 (16)
Years of experience		8 (13)
Other (Table S2)		18 (29)
***Number of specialties represented in the panel***	**69 (86)**	
Several		53 (77)
One		16 (23)
***Inclusion of multiple stakeholders*** c	**70 (88)**	
**Yes**		40(57)
**Types of stakeholders** c ^,^ d		
Healthcare professionals		38 (95)
Informal caregivers		25 (63)
Methodologists / Researchers / Public health experts		14(35)
Managers		11 (27)
Patients		8 (20)
Other		9 (22)
**No**		30 (43)
***Number of stakeholder types per study***		
One		1 (2)
Two		19 (48)
Three		14 (35)
Four		6 (15)
**Years of experience**	**7 (9)**	
*Median (Q1;Q3)*		15 (10;18)

a Percentage of studies that reported this item over the total number of studies (n = 80).

b All percentages were calculated relative to the total number reported.

c The total percentage may exceed 100% because some studies used more than one criterion.

d Percentages were calculated using 40 (number of studies with several stakeholders) as the denominator.

The number of individuals invited to participate was reported in 76/80 (95%) articles. Authors reported that they asked participants their willingness to participate to the survey before the first Delphi round in only 21/80 (26%) studies. Only 10/80 (13%) studies described the use of specific techniques to encourage participation, and there was no statistically significant difference in first-round response rates between studies where such techniques were reported and other studies (89.5% vs. 90.0%, *p* = 0.6).

### Data on the Progress of the Delphi Procedure

Of the 80 studies, 49 were modified Delphi procedures and 29 were basic Delphi procedures; procedure type was not specified in 2 articles (**TABLE 4**).

**Table 4:** Characteristics of the Delphi procedure.

Characteristics	Reported^a^n (%)	Present^b^n (%)
***Type of Delphi procedure***	**78 (98)**	
Modified		49 (63)
Basic		29 (37)
Not specified	2	
***Timing of the meeting in modified Delphi procedures (n = 49)***	**34 (69)**	
Between rounds		19 (56)
After the last round		15 (44)
***Number of rounds***		
***Delphi***	**23 (79)**	
*Median (Q1;Q3)*		3 (2;3)
*Min;Max*		2;4
**Modified Delphi**	**43 (88)**	
*Median (Q1;Q3)*		2 (2;3)
*Min;Max*		1;6
***Delphi procedure duration (weeks)***	**23 (29)**	
*Median (Q1;Q3)*		20 (8;28)
***Main methods used to send the questionnaires*** c	**45 (56)**	
Mail		35 (78)
Internet		10 (22)
Telephone/Fax		3 (7)
***Geographical scope***	**71 (89)**	
National		60 (85)
International		11 (15)
***Question formulation***	**73 (91)**	
Rating scale + Open question		47 (64)
Rating scale		22 (30)
Open question (comments)		1 (2)
Other		3 (4)
*Prioritize quality indicators*		2
*Remove and add quality indicators*		1
***Minimum value of rating scale*** * (n = 69)*		
Median(Q1;Q3)		1 (1;1)
***Maximum value of rating scale*** * (n = 69)*		
Median(Q1;Q3)		9 (5;9)
***Consensus methods***	**62 (77)**	
Median score+Percentage of agreement		22 (35)
Median score		10 (16)
Percentage of agreement		9 (15)
Rand method		8 (13)
IPR and IPRS method^d^		2 (3)
Other		8 (13)
Not clear		3 (5)
***Feedback***	48 (60)	
Quantitative		28 (58)
Quantitative and qualitative		19(40)
Qualitative		1 (2)
***Individual feedback reported***	**31 (39)**	

a Percentage of studies that reported this item over the total number of studies (n = 80).

b All percentages were calculated relative to the total number reported.

c The total percentage may exceed 100% because some studies used more than one criterion.

d IPR, intrapercentile range; and IPRS, intrapercentile range adjusted for symmetry.

The number of rounds was reported in 66/80 (83%) studies. The methods used to describe a consensus were not described in 18/80 (23%) studies and were unclear in 3/62 (5%) studies. Five main methods were used to achieve a consensus about the selected indicators. (a) In 22/62 (35%) studies, indicators with median scores above a predefined threshold and a high level of agreement among panel members were selected; an example is selection of indicators having a median score of 8 or more with 75% or more of the ratings being in the lowest or highest tertile. (b) In 10/62 (16%) studies, selection was based only on a median score greater than a predefined threshold (e.g., indicators having a median score of 7 or more were selected). (c) In 9/62 (15%) studies, the proportion of experts who rated the indicator within the highest region of the scale had to be greater than a predefined threshold (e.g., 75% or more of the experts giving the indicator scores of 7, 8, or 9). (d) In 8/62 (13%) studies, Rand UCLA criteria for agreement were used (for a 9-member panel using a 9-point Likert scale, no more than 2 members rate the indication outside the 3-point region (1–3; 4–6; 7–9) containing the median) [23]. (e) Finally, in 2/62 (3%) studies, indicator selection relied on the interpercentile range (IPR) and interpercentile range adjusted for symmetry (IPRS), with an IPR value greater than the IPRS value indicating that the indicator was rated with disagreement [23]. Concerning the methods used by organisers to send the response back to the panel, 40% (32/80) of studies didn't report that feed back to panel members was given between rounds and 61% (49/80) didn't report that own individual response were feed back to the panel.

### Data on Delphi Procedure Results

Response rates for all rounds were reported in only 39% (31/80) of studies. For these, the median response rate was 90% (Q1:80%–Q3:100% ) in the first round (87% for basic Delphi and 92% for modified Delphi studies) and 88% (Q1:69%–Q3:96%) in the last round(90% for basic Delphi and 87% for modified Delphi studies). The number of indicators selected at the end of the survey was reported in 68/80 (85%) articles, in which the median was 29 (Q1–Q3: 18–52.5). The lists of indicators were available in 69/80 (86%) reports but the final set of indicators was given in only 46/69 (67%) reports. The list of indicators included in the first questionnaire was available in 23/69 (33%) articles and additional information on selection in 8/69 (12%) articles (discarded indicators, 2 articles; sample of selected indicators, 2 articles; indicators included in the next round, 2 articles; indicators given high scores, 1 article; and indicators included after external peer review, 1 article). Finally, 37/80 (46%) articles included a flow chart of the indicators. A single study provided the Delphi questionnaires in an appendix.

## Discussion

We appraised the use and reporting of Delphi procedures for selecting healthcare quality indicators. We included 80 articles published as of December 2009. Most studies used a modified Delphi procedure with a physical meeting, usually between Delphi rounds. Considerable variability was noted across studies in the characteristics of the Delphi procedure. Moreover, study reports did not consistently provide details that are important for interpreting the results. For example, only 39% of studies reported that individual feedback was given between rounds and the method used to define a consensus was specified in only 77% of studies. Moreover, response rates for all rounds were reported in only 31% of studies. Information on both points is needed to evaluate the validity and credibility of the results. If the Delphi method is incompletely described this may affect the overall quality of the final consensus and the selected indicators are unlikely to gain the level of credibility needed for adoption in clinical practice. Our results are supported by those found by Sinha and colleague [24], who identified many variability in methodology and reporting of this method to select core outcomes in clinical trials.

To our knowledge, this is the first systematic review of the use and reporting of Delphi procedures for selecting healthcare quality indicators. The strengths of the study include the retrieval of studies published over a 30-year period (1978–2009) and the use of a standardized data extraction form based on data from a literature search. However, our study has limitations. No consensus exists on how to assess the applicability of a Delphi procedure. Consequently, we identified applicability items based on a literature review, and these items may vary in relevance. Several modifications of the original Delphi method have been described in the literature, but standardized definitions of these modifications are not available. We defined a modified Delphi procedure as Delphi rounds plus a physical meeting, in keeping with the definition given in most of the included articles. Finally, a single investigator screened all retrieved articles for eligibility and collected all the data. However, a quality assurance procedure was performed.

### Criteria Used to Select QI Depend on the Survey Objective

The Delphi technique has been used since the late 1970s for quality-indicators selection in the field of healthcare [25], [26]. Ideally, quality indicators would be based on evidence from rigorously conducted empirical studies. In practice, however, such evidence is rarely available in adequate amounts [27]. Therefore, quality indicators for healthcare must be selected partly or largely based on the opinions and experience of clinicians and others with knowledge of the relevant topic [28], [29].

In healthcare, several criteria are used to select indicators via the Delphi method. We found that the most commonly used criterion was validity. Validity is defined as the extent to which the characteristics of the indicator are appropriate for the concept being assessed. Generally, this criterion is used when the objective is to develop new indicators in a given field. Indicators selected via consensus methods such as the Delphi procedure have high face validity, which is a prerequisite for any quality indicator. However, validity is not enough and quality indicator should exhibit other characteristics and required metrological properties like any measuring instrument such as health measurement scales or analytical methods [30]. Indeed , an indicator is considered a good measure if it meets criteria including reliability, sensitivity, specificity, and feasibility (or applicability) [20], [31]. The common use of these characteristics can facilitate acceptance and implementation of indicators developed. For example, it has been shown that the validity and feasibility of a specific guideline predict implementation of the guideline in the clinical setting [32].

We noted that selection criteria changed between rounds in 13/73 (18%) studies. According to the rules of the Delphi procedure, the selection criteria should be the same in all rounds. Changing the selection criteria is the equivalent of conducting a distinct Delphi procedure, in which case achieving a consensus is extremely difficult.

### Simple Means to Increase Adhesion

Selection of the panel members may be crucial if the group consensus technique is to work properly [33]. Participants should be chosen based on their willingness to participate and knowledge of the relevant topic [34]. To maintain a high response rate throughout the Delphi procedure, participants should be asked whether they want to commit to the project. For instance, they could be sent an information letter explaining the method and the reasons their participation to the whole process would be necessary, as well as a form for collecting their consent to complete the entire Delphi process.

### A Heterogeneous Panel Member

Studies have shown that panel composition influences ratings [35]. Indeed, ratings vary across specialties [36] and between mixed and single-specialty panels [37], [38]. Studies in psychology [39] suggest that heterogeneity in a decision-making group may lead to better performance than homogeneity. To enhance the credibility and acceptance of quality indicators, the panel should reflect the full range of stakeholders who have an interest in the results of the study. Moreover, different stakeholders often have very different point of views about quality of care [40], which enrich the results of the Delphi procedure. Therefore, depending on the study objective, inclusion in the panel of healthcare-quality professionals, patients or patient representatives and methodologists should be encouraged. To obtain a panel that is representative of all stakeholders concerned by the study results, study design must specify the characteristics of the participants, such as gender, professional experience, education or employment.

The Delphi could be a long process. The participants must complete the questionnaire despite their busy schedules and non-respondents must be contacted. Duffield [33] reported that each round can take up to 8 weeks to complete. That is probably due to the need to follow up non respondents and the time needed to adequately analyze results to prepare feedback for the next round.

### Administration of Questionnaire: Which Ways?

Delphi questionnaires are usually sent by mail, although Internet-based questionnaires are being increasingly used [41]–[43] to save time and to increase dissemination. However, a study showed significantly lower response rates with Internet-based questionnaires than with mailed questionnaires [44]. Conceivably, using both mail and the Internet might improve questionnaire dissemination and increase response rates. Moreover, the advantage of the Delphi procedure is that experts who live and work far apart from each other can participate. However, we found that only 11/71 (13%) studies included participants from different countries. In 47/73 (64%) studies in our review, the panellists rated the indicators on a Likert scale (usually ranging from 1 to 9) and were able to make comments. This method allows panellists to explain their choices and to express their views on the indicators, thus supplying the investigators with useful information for developing the questionnaire of next rounds.

### Delphi vs Modified Delphi

Delphi participants are polled individually, usually via self-administered questionnaires with no physical meeting, over two or more rounds. After each round, the results are reported to the group.

In more than half the studies included in our review, at least one physical meeting of panel participants was held. Having a physical meeting contradicts one of the basic rules of the Delphi procedure, which is avoidance of situations that might allow one of the panel members to dominate the consensus process. Conversely, absence of a meeting may deprive the Delphi procedure of benefits related to face-to-face exchange of information, such as clarification of reasons for disagreements [45]. For example, other formal consensus methods such as the nominal group technique [46] and the Rand UCLA Appropriateness Method [23] use a highly structured meeting to gather information from relevant experts. Therefore, during the Rand UCLA Appropriateness Method meeting, no indications are discarded between rounds and, consequently, no potential information is lost. In the nominal group method, the meeting involves rating, discussing and, finally re-rating a series of items. A panel meeting at the end of the Delphi procedure may be useful when reaching a consensus is difficult. The best strategy would be a physical meeting at the end of the last round to exchange views and resolve uncertainties. However, the meeting should be well structured and should take place under favourable conditions(good surrounding and general environment) [47] with a moderator to contain the influence of dominant personalities. Studies on methods involving face-to-face interaction show that the way a meeting is structured and organized affects the group interactions [48] and influences the manner in which the group produces results.

### No Consensual Definition of “Consensus”

As previously mentioned, the most sensitive methodological issue with the Delphi method is the definition of a consensus among participants. The investigators must decide how agreement among participants will be measured and, if the agreement rate is used, what cut-off will be used to define a consensus. Our review shows that the method used to define a consensus varied across the included studies. The RAND researchers [23] definition was widely used, although in some cases the number of panellists was not a multiple of 9. In one study [49] involving two Delphi rounds, the agreement rate used to define a consensus was higher in the second round than in the first. In another study, the procedure was stopped when the last two rounds showed no significant difference in results as assessed using the Wilcoxon signed rank test [43]. Since as many rounds are held as needed to achieve a consensus or the ‘point of diminishing returns’, the criterion used to define a consensus influences the number of rounds. Stopping the Delphi procedure too soon may lead to results that are invalid or not meaningful, but a large number of rounds may cause participant fatigue with steep dropout rates [50]. The recommended number of rounds is two or three, in keeping with our results. However, there is very little scientific evidence on which to base decisions about the optimal number of rounds.

### Feedback between Rounds: Important Aspect of the Methodology

Feedback is an essential component of the Delphi procedure. Nevertheless, among studies that used a rating scale and open questions, only 19/35 (54%) reported both qualitative and quantitative feedback to the panel and even fewer reported individual feedback. It has been recommended that feedback should include qualitative comments and statistical measures [51]. After each round, each participant should be given the panel results (median, lowest, and highest ratings), the participant's response, and a summary of all comments received. These data inform each participant of his or her position relative to the rest of the group, thus assisting in decisions about replies during future Delphi rounds.

In conclusion, the Delphi procedure is valuable for achieving a consensus about issues where none existed previously. However, our findings indicate a need for improving the use and reporting of this technique. In **TABLE 5**, we outline practical guidance that may improve the optimal use and reporting of the Delphi method in quality indicator research. We are aware that the Delphi procedure is used in many other setting whether an appraisal of Delphi practices is also be performed. Nevertheless, our review provides helpful information on the use of Delphi in our field and additional research is needed to investigate its use in other setting. Also determining when to stop the Delphi procedure is a major issue. The optimal size and composition of the panel need to be determined. Authors must strive to provide sufficient detail on the method they use.

**Table 5:** Practical guidance for using and reporting Delphi procedures performed to select healthcare quality indicators (QIs).

Points to consider	Recommendations for planning and using the Delphi procedure	Recommendations for reporting the Delphi procedure
**Questionnaire for the first round**	Define the study objective and what you expect of participantsAre the selection criteria appropriate for the study objective? If the objective is to develop a new QI and to evaluate whether an indicator has the appropriate characteristics for the concept being assessed, use validity as the selection criterion. If the objective is to evaluate the availability in medical records of information relevant to a QI, use feasibility as the selection criterion.Use a 1–9 Likert rating scale and define the steps on the scale clearly (e.g., indicate what the lowest and highest ratings mean)Allow the panel to comment and to add QIs.Define consensus and criteria for stopping the Delphi procedure	Study objective, method for QI selection, number of QIs in the first questionnaire, criteria for QI selection, how questions were formulated, and definition of a consensus
**Experts**	Create a heterogeneous group of experts (healthcare professionals, informal caregivers, patients)Ask the potential panel participants about their willingness to participate; send an information letter explaining the Delphi procedure and benefits from participation; include an agreement form with the letter.Invite a very large number of experts, if possible from different countries.	Composition and characteristics of the panel, number of participants (diagram of participant flow), response rate for each round, whether special techniques were used to invite participants, and geographic scope of the Delphi procedure
**Sending questionnaires**	Use two methods (Internet and mail) to target as many people as possible and to increase the response rate	Report the method(s) used to send the questionnaires
**Next rounds**	Construct the next questionnaires based on the results of the preceding rounds.Exclude QIs for which there was no consensus.Send each participant a personalized questionnaire with:• quantitative group results (median, minimal, and maximal ratings)• qualitative feedback: abstract of panel members' comments• the participant's own response to illustrate position versus the group	Flow of QIs with the QIs eliminated and added at each round.Method used to inform the participants of the results of previous rounds
**Final round**	If agreement is reached among panel members: End the Delphi procedureWhen reaching a consensus is difficult or consensus is unclear, a physical meeting is recommended.	Report the number of rounds, whether a meeting was held (and if there was a meeting, what the participants did and who attended), duration of the Delphi procedure, results for each QI score and list of selected QIs.If possible, include a copy of the questionnaires in an appendix.

## Supporting Information

Appendix S1 Data extraction form.
(DOC)

Figure S1 Number of publications per year reporting Delphi procedures used to select quality indicators in healthcare.
(DOC)

Table S1 Other criteria used to select quality indicators.
(DOC)

Table S2 Other Criteria used to choose potential participant.
(DOC)

Box S1 Example of definition of selection criteria.
(DOC)

## References

[1] Institut of medicine: IOM definition of quality: http://www.iom.edu/Global/News%20Announcements/Crossing-the-Quality-Chasm-The-IOM-Health-Care-Quality-Initiative.aspx

[2] Kohn LTC, JM, Donaldson MS (1999). To Err is Human, Building a Safer Health System.

[3] Majeed FA, Voss S (1995). Performance indicators for general practice.. Bmj.

[4] Donabedian A (1980). Explorations in quality assessment and monitoring: the definition of quality and approaches to its assessment.

[5] Pringle M (1998). Preventing ischaemic heart disease in one general practice: from one patient, through clinical audit, needs assessment, and commissioning into quality improvement.. Bmj.

[6] Campbell SM, Cantrill JA (2001). Consensus methods in prescribing research.. J Clin Pharm Ther.

[7] Hearnshaw HM, Harker RM, Cheater FM, Baker RH, Grimshaw GM (2001). Expert consensus on the desirable characteristics of review criteria for improvement of health care quality.. Qual Health Care.

[8] Hasson F, Keeney S, McKenna H (2000). Research guidelines for the Delphi survey technique.. J Adv Nurs.

[9] Campbell SM, Cantrill JA, Roberts D (2000). Prescribing indicators for UK general practice: Delphi consultation study.. Bmj.

[10] Green B, Jones M, Hughes D, Williams A (1999). Applying the Delphi technique in a study of GPs' information requirements.. Health Soc Care Community.

[11] Fink A, Kosecoff J, Chassin M, Brook RH (1984). Consensus methods: characteristics and guidelines for use.. Am J Public Health.

[12] Powell C (2003). The Delphi technique: myths and realities.. J Adv Nurs.

[13] Jairath N, Weinstein J (1994). The Delphi methodology (Part one): A useful administrative approach.. Can J Nurs Adm.

[14] Adler M, Ziglio E (1996). Gazing into the oracle: the delphi method and its application to social policy and public health.. Jessica Kingsley Publisher.

[15] Roland M, Holden J, Campbell S (1998). Quality assessment for general practice: supporting clinical governance in primary care groups.

[16] Beattie E, Mackway-Jones K (2004). A Delphi study to identify performance indicators for emergency medicine.. Emerg Med J.

[17] Normand SL, McNeil BJ, Peterson LE, Palmer RH (1998). Eliciting expert opinion using the Delphi technique: identifying performance indicators for cardiovascular disease.. Int J Qual Health Care.

[18] Keeney S, Hasson F, McKenna H (2006). Consulting the oracle: ten lessons from using the Delphi technique in nursing research.. J Adv Nurs.

[19] Grol R, Baker R, Moss F (2004). Quality improvement research: understanding the science of change in health care.. BMJ Books.

[20] Mainz J (2003). Defining and classifying clinical indicators for quality improvement.. Int J Qual Health Care.

[21] Gagliardi AR, Fung MF, Langer B, Stern H, Brown AD (2005). Development of ovarian cancer surgery quality indicators using a modified Delphi approach.. Gynecol Oncol.

[22] Esmaily HM, Savage C, Vahidi R, Amini A, Zarrintan M (2008). Identifying outcome-based indicators and developing a curriculum for a continuing medical education programme on rational prescribing using a modified Delphi process.. BMC Med Educ.

[23] Fitch K, Bernstein S, Aguilar M, Burnand B, LaCalle J

[24] Sinha IP, Smyth RL, Williamson PR Using the Delphi technique to determine which outcomes to measure in clinical trials: recommendations for the future based on a systematic review of existing studies.. PLoS Med.

[25] Starkweather DB, Gelwicks L, Newcomer R (1975). Delphi forecasting of health care organization.. Inquiry.

[26] Grimes RM, Moseley SK (1976). An approach to an index of hospital performance.. Health Serv Res.

[27] Chassin M, Hopkins A (1989). How do we decide whether an investigation or procedure is appropriate?,. Appropriate investigation and treatment in clinical practice.

[28] Agency for Health Care Policy and Research (1995). AHCPR Clinical Practice Guideline Program..

[29] Mann T (1996). Clinical guidelines. Using clinical guidelines to improve patient care in the NHS.

[30] Stereiner DL, Norman GR (1995). Health measurement scales: a pratical guide to their development and use.

[31] Rubin HR, Pronovost P, Diette GB (2001). From a process of care to a measure: the development and testing of a quality indicator.. Int J Qual Health Care.

[32] Grol R (2001). Successes and failures in the implementation of evidence-based guidelines for clinical practice.. Med Care.

[33] Duffield C (1993). The Delphi technique: a comparison of results obtained using two expert panels.. Int J Nurs Stud.

[34] Goodman CM (1987). The Delphi technique: a critique.. J Adv Nurs.

[35] Campbell SM, Hann M, Roland MO, Quayle JA, Shekelle PG (1999). The effect of panel membership and feedback on ratings in a two-round Delphi survey: results of a randomized controlled trial.. Med Care.

[36] Kahan JP, Park RE, Leape LL, Bernstein SJ, Hilborne LH (1996). Variations by specialty in physician ratings of the appropriateness and necessity of indications for procedures.. Med Care.

[37] Coulter I, Adams A, Shekelle P (1995). Impact of varying panel membership on ratings of appropriateness in consensus panels: a comparison of a multi- and single disciplinary panel.. Health Serv Res.

[38] Leape LL, Park RE, Kahan JP, Brook RH (1992). Group judgments of appropriateness: the effect of panel composition.. Qual Assur Health Care.

[39] Bantel K (1993). Comprehensiveness of strategic planning: the importance of heterogeneity of a top team.. Psychol Rep.

[40] Hong CS, Atlas SJ, Chang Y, Subramanian SV, Ashburner JM Relationship between patient panel characteristics and primary care physician clinical performance rankings.. Jama.

[41] Colucci E, Kelly CM, Minas H, Jorm AF, Chatterjee S Mental Health First Aid guidelines for helping a suicidal person: a Delphi consensus study in India.. Int J Ment Health Syst.

[42] Bisson JI, Tavakoly B, Witteveen AB, Ajdukovic D, Jehel L TENTS guidelines: development of post-disaster psychosocial care guidelines through a Delphi process.. Br J Psychiatry.

[43] Hejblum G, Ioos V, Vibert JF, Boelle PY, Chalumeau-Lemoine L (2008). A web-based Delphi study on the indications of chest radiographs for patients in ICUs.. Chest.

[44] Leece P, Bhandari M, Sprague S, Swiontkowski MF, Schemitsch EH, Tornetta P (2004). Internet versus mailed questionnaires: a controlled comparison (2).. J Med Internet Res.

[45] Walker A, Selfe J (1996). The Delphi method: a useful tool for the allied health researcher.. nternational Journal of Therapy and Rehabilitation.

[46] Van de Ven A, Delbecq A (1972). The nominal group as a research instrument for exploratory health studies.. Am J Public Health.

[47] Reagan-Cirincione P, Rohrbaugh J (1992). Decision Conferencing A Unique Approach to the Behavioral Aggregation of Expert Judgment Part II.

[48] Pavitt C (1993). What (little) we know about formal group discussion procedures: A review of relevant research.. Small Group Research.

[49] Shield T, Campbell S, Rogers A, Worrall A, Chew-Graham C, Gask L (2003). Quality indicators for primary care mental health services.. Qual Saf Health Care.

[50] Schmidt R (1997). Managing Delphi surveys using nonparametric statistical techniques.. Decision Sciences.

[51] Murphy MK, Black NA, Lamping DL, McKee CM, Sanderson CF (1998). Consensus development methods, and their use in clinical guideline development.. Health Technol Assess.

[52] Holloway RG, Vickrey BG, Benesch C, Hinchey JA, Bieber J (2001). Development of performance measures for acute ischemic stroke.. Stroke.

[53] McGory ML, Shekelle PG, Ko CY (2006). Development of quality indicators for patients undergoing colorectal cancer surgery.. J Natl Cancer Inst.

[54] Wang CJ, McGlynn EA, Brook RH, Leonard CH, Piecuch RE (2006). Quality-of-care indicators for the neurodevelopmental follow-up of very low birth weight children: results of an expert panel process.. Pediatrics.

