# Supplementary material for: Using and Reporting the Delphi Method for Selecting Healthcare Quality Indicators: A Systematic Review
Source: PLoS One. 2011 Jun 9;6(6):e20476. doi: 10.1371/journal.pone.0020476 (PMC3111406; doi:10.1371/journal.pone.0020476)
Supplement: Appendix S1 — Data extraction form. (DOC) [file pone.0020476.s001.doc]

**Appendix S1**: Data extraction form

**Using the Delphi method for selecting quality indicators**

Reader

Article

**Number**  |__|__|__|

**Title** …………………………………………………………………………………………………………………………………………………………………………………………………………………

**First author** …………………………………………………..

**Journal** …………………………………………………………..

**Date of publication** |__|__|__|__|

**Area** …………………………………………………………………

***Preparation of the first Delphi questionnaire***

1. **Type of quality indicators**

🞎 Outcome indicators (*refer to the impact on the health status of patients or populations*)

🞎 Structure indicators (*refer to static or technical aspects of care (e.g., attributes of service providers or organizational characteristics*)

🞎 Process indicators (*refer to steps taken in caring for the patient*)

🞎 Other……………….

🞎 Not reported

1. **Primary selection of quality indicators included in the questionnaire**

🞎 Literature search (Medline, Cochrane…)

🞎 Guidelines

🞎 Development of new measure (*if researchers developed new quality indicators they intended to validate*)

🞎 Internal consensus (*For example, consultation between researchers and survey organizers)*

🞎 Other……………….

🞎 Not reported

1. **Quality indicators were selected based on**

🞎 Validity *(the extent to which quality indicator has the appropriate characteristics for the concept being assessed)*

🞎 Acceptability (*is the indicator acceptable to both those being assessed and those undertaking the assessment?)*

🞎 Feasibility (*if data were available and collectable*)

🞎 Clarity

🞎 Agreement (agree/not agree)

🞎 Other…………

🞎 Not reported

1. **Criteria were defined?**

🞎 Yes

🞎 No

1. **Did criteria change between rounds?**

🞎 Yes

🞎 No

🞎 Not reported

1. **If so, what list of criteria used in the next rounds:**

- 2^nd^ round………………….
- 3rd round …………………
- 4th round …………………

1. **Number of quality indicators included initially** |__|__|__|

🞎 Not reported

1. **Additional quality indicators added between rounds**

🞎 Yes

🞎 No

***Participants***

1. **Participants were first asked about their willingness to participate to the study. The questionnaire was sent only to participants who stated they were willing to participate in the study**

🞎 Yes

🞎 Not reported

1. **Number of potential participants invited** |__|__|__|
2. **How were the potential participants chosen?**

🞎 Years of experience

🞎 Renown

🞎 Recommendation

🞎 Members of an organization

🞎 Random

🞎 Other

🞎 Not reported

1. **Were the participants described (e.g., specialty, age, sex, ..).**

🞎 Yes

🞎 No

1. **Years of experience of the participants**

🞎 Reported

🞎 Not reported

**If reported, how many mean years of experience** |__|__| years

1. **Were the panel members in a single specialty or multiple specialties?**

🞎 One specialty

🞎 Multiple specialties

🞎 Not reported

1. **Inclusion of multiple stakeholders**

🞎 Yes

🞎 No

🞎 Not reported

**If yes, type of stakeholders**,

🞎 Patients

🞎 Healthcare professionals

🞎 Managers

🞎 Informal caregivers

🞎 Other………………..

***Delphi procedure***

1. **Type of Delphi procedure**

🞎 Basic (self-administered questionnaires, sent by any means, with no meeting)

🞎 Modified (in addition to the questionnaires, physical meeting to discuss results or to rate indicators)

1. **If modified Delphi procedure, when did the meeting occur?**

🞎 Before the first round

🞎 After the last round

🞎 Between rounds

🞎 Not reported

🞎 Other

**What did the participants do during the meeting?**

🞎 Rate quality indicators

🞎 Discuss quality indicators

🞎 Both

🞎 Other……..

1. **Number of rounds** |__|__| Not reported 🞎
2. **Duration of the Delphi procedure**

|__|__|__| Weeks

|__|__| Months

|__|__| Years

🞎 Not reported

1. **Geographic scope**

🞎 Nationwide survey

🞎 International survey

🞎 Not reported

1. **How were the potential participants invited?**

🞎 Mail

🞎 Internet

🞎 Both

🞎 Other……………………….

🞎 Not reported

1. **Were special techniques used to encourage participation?** (*For example: reminder or stamped addressed reply envelopes*)

🞎 Yes

🞎 Not reported

1. **Questionnaire was available in the article or an appendix**

🞎 Yes

🞎 No

1. **Question format**

🞎 Open question (comments)

🞎 Rating of indicators on a scale (Likert Scale)

🞎 Both

🞎 Other………….

🞎 Not reported

1. **Was there a rating scale?**

🞎 Yes

🞎 No

**If Yes, what were the lowest and highest possible ratings** From |__| to |__|__|

**Was the scale clearly defined? For example, were the meanings of the lowest and highest ratings defined?**

🞎 Yes

🞎 No

🞎 Not reported

1. **Feedback** *(How organizers sent the responses back to the panel)*

🞎 Quantitative *(Statistical summaries illustrating the collective opinion such as central tendency, variance, median, and minimal and maximal ratings)*

🞎 Qualitative *(Abstract of panel comments)*

🞎 Both

🞎 Not reported

1. **Individual feedback** *(Organizers sent each panel member his or her own response)*

🞎 Reported

🞎 Not reported

🞎 No

1. **How was consensus obtained?**

Round 1:

Round 2:

Round 3:

Round 4:

🞎 Not reported

🞎 Not clear

***Results***

1. **Number of quality indicators selected at the end of the procedure** |__|__|__|

🞎 Not reported

1. **Percentage or number of respondents**

- 1st round |__|__|__|
- 2^nd^ round |__|__|__|
- 3rd round |__|__|__|
- 4th round |__|__|__|
- 🞎 Not reported

1. **Flow chart or description of QI flow**

🞎 Yes

🞎 No

1. **List of quality indicators**

🞎 Yes

🞎 No

**If yes, which quality indicators**

🞎 All quality indicators included in the first round

🞎 Only quality indicators selected at the last round

🞎 Both

🞎 Other………………………………..
