# Supplementary material for: Using and Reporting the Delphi Method for Selecting Healthcare Quality Indicators: A Systematic Review
Source: PLoS One. 2011 Jun 9;6(6):e20476. doi: 10.1371/journal.pone.0020476 (PMC3111406; doi:10.1371/journal.pone.0020476)

**Figure S1**: Number of publications per year reporting Delphi procedures used to select quality indicators in healthcare


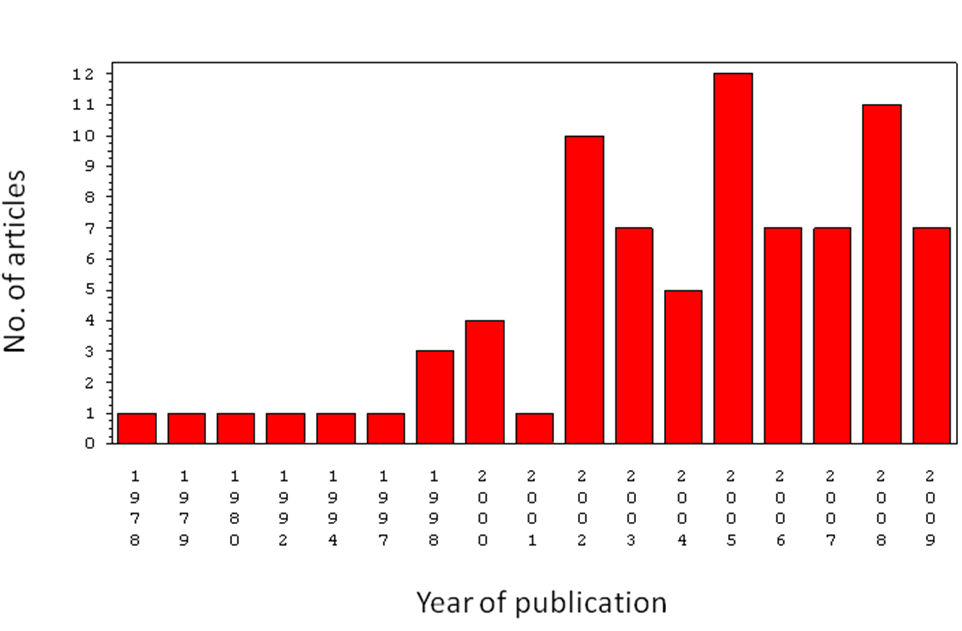

Supplement: Figure S1 — Number of publications per year reporting Delphi procedures used to select quality indicators in healthcare. (DOC) [file pone.0020476.s002.doc]
