# Supplementary material for: Using and Reporting the Delphi Method for Selecting Healthcare Quality Indicators: A Systematic Review
Source: PLoS One. 2011 Jun 9;6(6):e20476. doi: 10.1371/journal.pone.0020476 (PMC3111406; doi:10.1371/journal.pone.0020476)
Supplement: Table S1 — Other criteria used to select quality indicators. (DOC) [file pone.0020476.s003.doc]

**Table S1 Other criteria used to select quality indicators**

| Criteria |
| --- |
| Meaningfulness  Relevance  Usefulness  Clarity  Appropriateness  Is it a real quality indicator? To identify whether you consider each scenario fits the definition of QI given in the questionnaire |
| Room for improvement |
| Impact on outcome |
| Interpretability |
| Action-ability |
| Necessity |
| Scientific acceptability |
| Applicability |
| Needed to evaluate primary care |
| Scientific soundness |
| Importance of performance assessment |
| Opportunity for improvement? whether the processes or outcomes measured are within the control of an Emergency Department or hospital |
| Sensitivity to nursing care |
| Prevent outcome |
| Adequacy of risk adjustment |
| Captures quality |
| Essentiality. That is, whether an item would be part of the routine care for most patients with any of these conditions (measured by a seven-part scale, from "essential" to "completely unnecessary"), |
| Association with quality of care |
| Scientific evidence |
| Clinical relevance |
| Prioritization |
| Measurability |
| Reproducibility |
| Pertinence |
| Indicator reflects high quality of general practice |
| Measure is recommended |
