# Supplementary material for: Using and Reporting the Delphi Method for Selecting Healthcare Quality Indicators: A Systematic Review
Source: PLoS One. 2011 Jun 9;6(6):e20476. doi: 10.1371/journal.pone.0020476 (PMC3111406; doi:10.1371/journal.pone.0020476)
Supplement: Table S2 — Other Criteria used to choose potential participant. (DOC) [file pone.0020476.s004.doc]

**Table S2** : Other Criteria used to choose potential participant

| **Other Criteria n=14** |  |
| --- | --- |
| Willing to participate + volunteered | 4 |
| Random | 4 |
| Chosen by a public agency or organization | 4 |
| Availability | 3 |
| Interest in search | 3 |
| Geographical location | 2 |
| Specific criterion such as age, language, or knowledge | 1 |
