# Supplementary material for: Using and Reporting the Delphi Method for Selecting Healthcare Quality Indicators: A Systematic Review
Source: PLoS One. 2011 Jun 9;6(6):e20476. doi: 10.1371/journal.pone.0020476 (PMC3111406; doi:10.1371/journal.pone.0020476)
Supplement: Box S1 — Example of definition of selection criteria. (DOC) [file pone.0020476.s005.doc]

**Validity**:

- an indicator was considered valid if sufficient scientific evidence existed to support a link between performance of that measure and overall positive outcomes to patients”(52)
- an indicator would be defined as valid if adequate scientific evidence or professional consensus exists to support a link between the performance of care specified by the indicator and the accrual of health benefits to the patient, and a physician or facility with higher rates of adherence to the indicator would be considered a higher-quality provider (and those with lower rates of adherence would be considered poorer-quality providers)”(53)

**Feasibility**:

- “A measure is considered feasible if information needed to assess adherence is thought to be available in the medical record or from patient or proxy surveys or interviews and likely to be accurate”(52)
- “A high feasibility score was given if the average medical record is likely to contain information that is needed to determine adherence, estimates of adherence on the basis of medical record data are likely to be reliable, and failure to document information that is relevant to the indicator is itself a marker of poor quality”(54)

**Box**: Example of definition of selection criteria
